# 3D Printing of Hierarchical Scaffolds Based on Mesoporous Bioactive Glasses (MBGs)—Fundamentals and Applications †

**DOI:** 10.3390/ma13071688

**Published:** 2020-04-04

**Authors:** Francesco Baino, Elisa Fiume

**Affiliations:** Applied Science and Technology Department, Institute of Materials Physics and Engineering, Politecnico di Torino, Corso Duca degli Abruzzi 24, 10129 Torino, Italy; elisa.fiume@polito.it

**Keywords:** biomaterials, bioglass, scaffold, mesoporous, additive manufacturing, tissue regeneration, hierarchical, porosity, drug delivery, ion release, bioactivity

## Abstract

The advent of mesoporous bioactive glasses (MBGs) in applied bio-sciences led to the birth of a new class of nanostructured materials combining triple functionality, that is, bone-bonding capability, drug delivery and therapeutic ion release. However, the development of hierarchical three-dimensional (3D) scaffolds based on MBGs may be difficult due to some inherent drawbacks of MBGs (e.g., high brittleness) and technological challenges related to their fabrication in a multiscale porous form. For example, MBG-based scaffolds produced by conventional porogen-assisted methods exhibit a very low mechanical strength, making them unsuitable for clinical applications. The application of additive manufacturing techniques significantly improved the processing of these materials, making it easier preserving the textural and functional properties of MBGs and allowing stronger scaffolds to be produced. This review provides an overview of the major aspects relevant to 3D printing of MBGs, including technological issues and potential applications of final products in medicine.

## 1. Introduction

Bioactive glasses (BGs) were invented by Prof. Larry L. Hench at the University of Florida (US) in 1969 [1,2]. Originally, the “bioactivity” of these materials referred to their unique capability of bonding to living bone through establishing a tight material/tissue interface while stimulating the expression of osteogenic factors in bone cells, responsible for the synthesis of new bone [3,4]. It was observed that, both *in vitro* and *in vivo*, chemical reactions mediated by ion exchange mechanisms occur between the surface of BGs and the surrounding biological fluids, which ultimately leads to the formation of a layer of nano-crystalline hydroxyapatite (HA) at the bone/implant interface [5]. This newly-formed phase is very similar to the calcium phosphate mineral phase (bioapatite) of bones in mammals [6]. Recently, some special BG compositions were also found potentially suitable for applications in contact with soft tissues, such as peripheral nerve repair [7] and wound healing [8].

The first BG developed by Hench belonged to a silicate system obtained by traditional melt-quenching process [9]. It was characterized by a quaternary oxide composition (45SiO_2_-6P_2_O_5_-24.5Na_2_O-24.5CaO wt.%) and has been commercially available on the market since 1984 with the tradename of 45S5 Bioglass^®^; since then, the biomaterials community has been engaged in a stimulating research activity on BGs aimed at further improving the promising results already obtained in healthcare [10]. 

The 45S5 Bioglass^®^ composition, as well as most of silicate BGs produced by traditional melt-quenching route, are affected by some inherent limitations including high processing temperatures required for casting (>1500 °C) and unavoidable devitrification upon sintering, which resulted in a decrease of the bioactive properties [11]. Another important issue is the strong relationship existing between BG composition and HA-forming ability. In melt-derived silicate systems, high amounts of SiO_2_ (>60 mol.%) suppress the HA-forming ability of the material and, hence, the bone-bonding mechanism. The reason behind this behavior is that the higher the amount of SiO_2_, the higher the chemical stability of the glass network. As a result, there is a decrease of BG dissolution rate, ionic release and exposure of hydroxyl (—OH) groups, which act as a nucleation site for HA on the glass surface [12]. 

Apart from being produced by traditional melt-quenching, BGs can also be synthesized by the sol-gel process. Such BGs, which began to be studied in the early 1990s, exhibit dramatically appealing features for bone tissue engineering applications [13]. Sol-gel synthesis is commonly defined as a chemical processing technique based on three steps: (i) preparation of a sol, that is, a precursor-containing colloidal solution, (ii) gelation of the sol and (iii) removal of the solvent by thermal treatment [14]. From a technological viewpoint, the major advantage of sol-gel materials over melt-derived materials is that high temperatures are not required in a wet synthesis, since the formation of the gel occurs at room temperature due to hydrolysis and poly-condensation reactions catalyzed by a proper chemical agent [15]. Interestingly, gel-derived BGs exhibit a wider compositional range allowing bioactivity and SiO_2_ can be included in the system up to 90 mol.% without any suppression of HA formation or significant decrease of the relevant reaction kinetics [13,16]. An explanation of this attractive property is that, unlike melt-derived BGs, sol-gel materials are typically characterized by an inherent mesoporosity and higher surface area (>50 m^2^/g vs. less than 1 m^2^/g), which boosts the ion-exchange mechanisms, allowing faster deposition of HA [17,18,19]. 

A polymeric template can be introduced in the sol-gel process as a structure directing agent (SDA) to finely tailor the textural properties of the glasses, like mesopore size, distribution and orientation. In “traditional” sol-gel materials (without SDA), the distribution of mesopores is disordered, quite broad and pore size can largely vary between 2 and 50 nm [20]. 

Owing to the presence of an inherent mesoporosity, gel-derived glasses are typically affected by lower mechanical properties compared to melt-derived ones, which has limited their application to the repair of non-load-bearing osseous sites [13]. To the best of the authors’ knowledge, to date only one gel-derived mesoporous glass composition (70SiO_2_–30CaO mol.%) reached the market and was cleared for clinical application in bone repair (TheraGlass^®^, MedCell, Burgess Hill, UK) [21]. The interested reader is referred to other recent review papers on BGs and related clinical applications [22,23]. 

The introduction of additive manufacturing technologies in the field of BGs and, especially, gel-derived BGs is showing great promise for expanding the applications of these materials and overcoming the barriers to clinical translation in the future. This “hot” research field is explored in the present review, which was prepared by carrying out a literature search on the database SCOPUS using the keywords “mesoporous,” “bioactive glass,” “scaffold,” “3D printing” and their combinations.

## 2. Mesoporous Bioactive Glasses (MBGs): An Overview

Over the last two decades, ordered mesoporous materials, characterized by high surface area and well-organized mesoporous texture, have been gaining increasing scientific interest in biomedicine. Various types of mesoporous silicas (e.g., SBA-15, MCM-41) were initially investigated and proposed as carriers for drug delivery applications since an ordered mesostructured texture allows a better control on release kinetics, thus making the therapeutic treatment more effective and confined to the target area [24,25,26,27,28]. Incorporation of additional oxides in the composition (e.g., CaO and P_2_O_5_) allowed obtaining mesoporous bioactive glasses (MBGs) [29,30,31], which are potentially able to provide multiple therapeutic actions by supporting bone tissue ingrowth and regeneration while simultaneously providing a local treatment of diseased bone through the controlled release of organic (drugs, growth factors) or inorganic therapeutic agents (metallic ions) [32,33,34,35]. The impressive versatility of these systems allowed scientists to design them, for example, as multipurpose biomaterials for improving bone healing, eliciting antiseptic action by introducing specific cations into the MBG structure (e.g., Ag^+^, Zn^2+^, Cu^2+^, Ga^2+^, Ce^3+^ and Ce^4+^) [36,37,38] or even targeting to tumor therapy [39]. In this regard, it has been demonstrated that including small amounts of Fe_2_O_3_ in MBGs is sufficient to induce a magnetic response of the material after exposure to an external magnetic field, which results in a localized production of heat [40]—this effect can lead to the selective death of cancer cells by hyperthermia [39].

**Figure 1:**
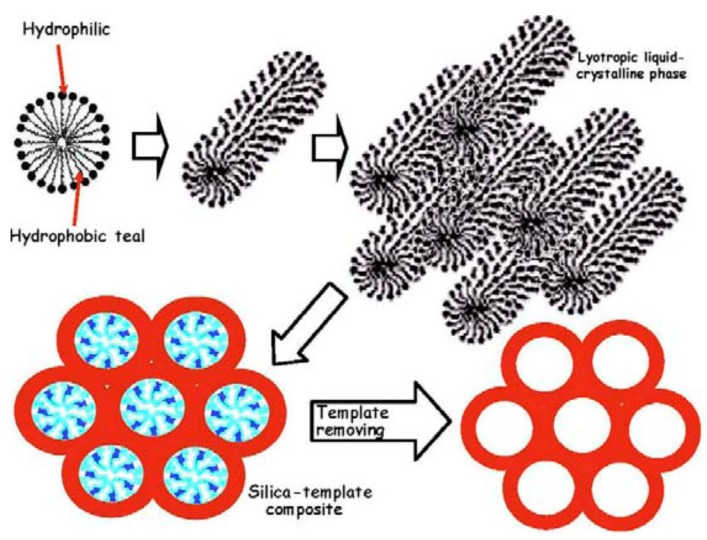
Schematic illustration of the steps leading to a mesoporous silicate material starting from a micellar solution: micelles from structure directing agent (SDA) link the hydrolyzed silica precursors through the hydrophilic component and self-assembly to form an ordered mesophase. Pictures reproduced from Arcos and Vallet-Regi [16] with permission.

**Figure 2:**
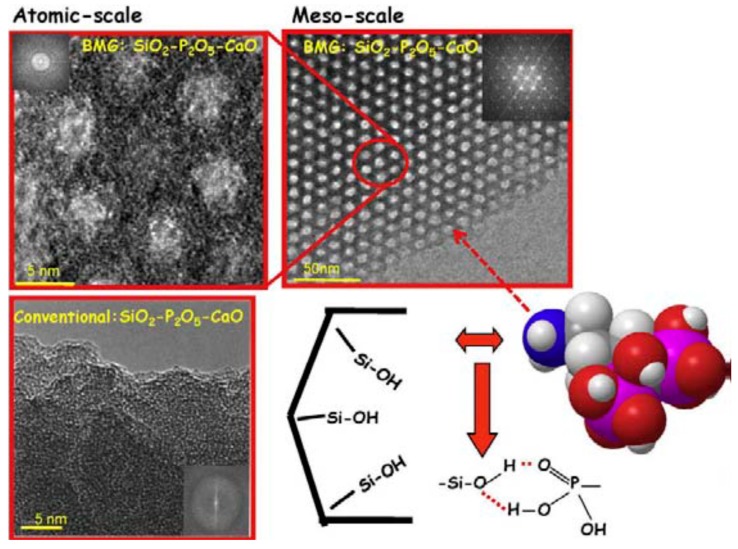
High-resolution transmission electron microscope (TEM) images of a silicate SDA-templated glass (MBG) (top) and a “conventional” sol-gel glass (without SDA) (bottom). Mesopores in the mesoporous bioactive glass (MBG) are well ordered according to a hexagonal symmetry. A schematic representation of alendronate molecule and its interaction with silanol groups at the mesopore surface are also displayed. Pictures reproduced from Arcos and Vallet-Regi [16] with permission.

The production of MBGs relies upon sol-gel synthesis combined with supramolecular chemistry [41]. It is worth pointing out that a disordered mesoporosity is inherently present in bioactive glasses produced *via* the sol-gel process in the absence of SDAs [13]. SDAs, acting as mesostructure templates (Figure 1) [16], can be employed to properly tailor the size, distribution and geometrical arrangement of the mesopores [20]. Looking at most of the existing literature, the definition “MBG” is conventionally referred to the latter type of sol-gel BGs (with SDA); Figure 2 [16] provides a further clarification of these issues. 

Some of the most commonly-used SDAs are cetyltrimethyl ammonium bromide (CTAB) and nonionic surfactants, such as Pluronic P123 and F127. Under specific and controlled pH and temperature, surfactant molecules are able to self-organize and create the mesoporous texture through the so-called evaporation-induced self-assembly process (EISA) [16]. In a typical EISA route [41], the molecules of the surfactant self-organize into micelles by exposing hydrophilic heads to the synthesis solution where the glass precursors (e.g., alkoxides or salts) have been added. The so-obtained mixture is then subjected to a thermal treatment (drying) for the complete removal of solvent; high-temperature treatments (calcination) are eventually necessary to remove SDA, precursor residues (e.g., nitrates) and consolidate the material [42].

**Figure 3:**
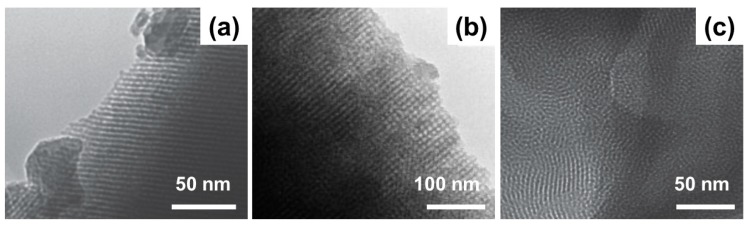
TEM images of mesoporous structures obtained by using P123 (**a**), F-127 (**b**) and cetyltrimethyl ammonium bromide (CTAB) (**c**) as SDAs in MBGs. Pictures reproduced from Wu et al. [33] and Yun et al. [44] with permission.

Different mesoporous textures can be obtained depending the type of surfactant used [43,44]; TEM images showing the MBG meso-structures resulting from the use of P123, F-127 and CTAB are displayed in Figure 3 [33,44].

CTAB is known to induce the formation of smaller mesopores (2–3 nm) compared to P123 and F-127 (4–10 nm). Worm-like morphology is typically generated by F-127, while 2D hexagonal mesoporosity was observed with P123 [32,41]. 

MBGs can be processed in different forms, including micro-/nano-particles with irregular or spherical shape, fibers, composite materials and 3D scaffolds; these products can be obtained by combining EISA method with other processing techniques (e.g., electrospinning for the production of fibers, hydrothermal methods or spray-drying for the production of micro- and nano-spheres) [45].

Fabrication of hierarchical MBG-based scaffolds is a challenge—in fact, mesopore size is almost three orders of magnitude lower than that of osteoblasts and, therefore, macroporosity must be somehow introduced in the final product in order to allow cell colonization and tissue in-growth [46]. 

**Figure 4:**
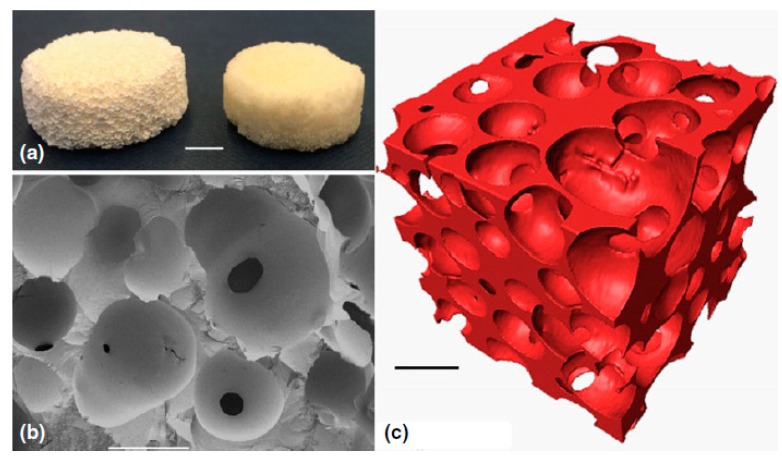
Sol-gel BG foam scaffold: (**a**) photographs after sintering at 600 °C (left) and 800 °C (right), scale bar = 5 mm; (**b**) Scanning electron microscope (SEM) image of a fracture surface, scale bar = 500 μm; (**c**) X-ray microtomographic reconstruction of the scaffold, scale bar = 500 μm. Pictures reproduced from Poologasundarampillai [49] with permission.

Actually, the first study on the development of hierarchical BG scaffolds was reported by Sepulveda et al. [47], who applied the foaming technique to the sol. This process involves the acid-catalyzed preparation of a sol by mixing together alkoxides and a catalyst. Then, a gelling agent (e.g., HF) and a surfactant are added to the sol that is foamed by vigorous agitation (i.e., air bubbles are introduced into the sol). As soon as the viscosity increases, the foam is poured immediately before gelation into molds; then, it is aged, dried and thermally stabilized (calcination). The obtained structure is hierarchical with both interconnected spherical macropores (deriving from foaming) and mesopores (which are inherent of the sol-gel process). It is interesting to highlight that, in this method, the surfactant somehow acts as a forming agent of macropores (Figure 4) The foaming process and calcination conditions were properly optimized to increase the compressive strength of these scaffolds to 4.5 MPa [48,49].

**Figure 5:**
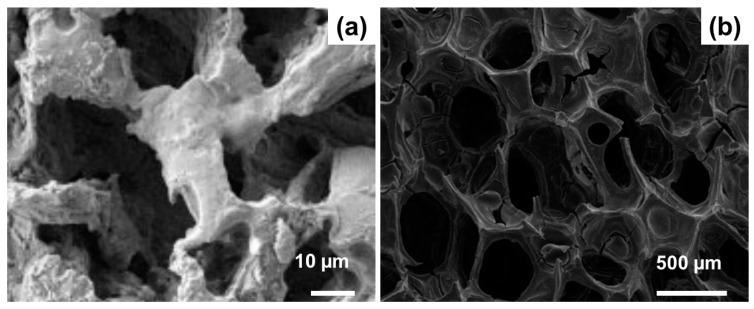
SEM images of MBG scaffolds for bone tissue engineering applications produced by porogen method (**a**) and replication of a polyurethane foam (**b**). Pictures reproduced from Yun et al. [44] and Wu et al. [40] with permission.

The foaming method, however, is unsuitable for obtaining hierarchical scaffolds based on MBG, where the sol already incorporates a surfactant acting as an SDA. The easiest strategies used to fabricate 3D MBG scaffolds include powder-based methods. MBG particles of proper size can be simply obtained by milling and sieving the mesoporous materials resulting from the EISA process followed by calcination. Among the techniques used for MBG scaffold production, the replication of macroporous templates [40,50,51,52,53] and the porogen methods [44] are perhaps the most popular. Typical morphologies given by these strategies are displayed in Figure 5 [40,44].

Foam replica method is considered one of the most versatile and effective techniques to obtain porous ceramic or glass scaffolds with bone-like trabecular structure exhibiting high porosity (>90 vol.%) and satisfactory pore interconnectivity [54,55]. Over time, apart from industrial polyurethane sponges, other macroporous templates have been proposed for making scaffolds, including natural structures derived from plants and vegetables [56]. However, scaffolds produced by replication methods are usually affected by ultralow mechanical strength (<50 kPa in compression [40]), which precludes the actual possibility of using the device for a safe treatment *in vivo* even in non-load-bearing sites. 

Higher mechanical performances can be achieved by applying porogen methods, in which polymeric spheres or particles (e.g., methyl cellulose [44]) are used as a macropore-forming agent that is thermally removed upon sintering. The main drawback of these techniques is the low degree of interconnectivity between adjacent pores and the relatively low volume of void space, which, combined with an inefficient control on porosity homogeneity, may preclude cell migration, fluid perfusion and vascularization after implantation. As a result, in recent years researchers’ attention has moved to additive manufacturing technologies for processing ceramic and glasses more effectively [57]. These techniques represent a valuable strategy to overcome most of the existing shortcomings of traditional fabrication methods for glass and ceramic scaffolds, such as low control on macropore architecture, low compressive strength and difficult reproducibility; on the other hand, they allow customization of the scaffold (patient-centered personalized medicine).

## 3. Fundamentals of 3D Printing and Application to BGs

In the last decade, several research groups intensively applied additive manufacturing technologies to process BGs in the attempt to obtain mechanically-resistant scaffolds with tailored and highly-controlled structural features. 

This broad class of fabrication techniques include laser-assisted methods (e.g., selective laser sintering, stereolithography) and controlled dispensing procedures (often simply called 3D printing). Selective laser sintering employs a laser beam to sinter thin layers of powdered materials to form 3D objects. The laser beam is scanned over the powder bed following a computer-aided design (CAD) model, thus raising the temperature of powders only in selected areas. Particles melt together and subsequent layers can be built directly on the top of the previously-sintered material [58].

Stereolitography uses a blend of glass or ceramic powders and a photocurable monomer. A UV laser beam, which cures the monomer, is selectively scanned over the surface of the blend following the cross-sectional profiles of the CAD model; subsequent layers are built directly on top of previously-cured layers with new layers of blend being deposited. After this step, non-polymerized material is removed, and sintering is performed to obtain the final product [59].

However, both these strategies require high investment costs to buy the proper equipment; therefore, the use of relatively affordable 3D printers is often preferred in a lab-scale scenario. The use of particles below 30 µm and extrusion nozzles between 100–580 µm allows obtaining 3D-printed BG structures with pores size ranging from few tens of micrometers to half a millimeter, which are potentially suitable for bone tissue engineering applications [57]. 

Generally, total porosity of 3D-printed scaffolds is lower than that obtained by foam replication and ranges within 50 vol.%–60 vol.%, which corresponds to the lower range of trabecular bone [6]. As a result, high mechanical properties can be achieved—such scaffolds can exhibit a compressive strength higher than 13 MPa and are then potentially able to match–to some extent–the compressive resistance of both trabecular and cortical bone [60]. Three-dimensional printing technology allows a relatively easy fabrication of scaffolds with different patterns characterized by a wide variety of pore sizes and shapes. BG scaffolds with porosity gradient or multiscale porosity have great potential for interfacial tissue engineering—for example, the interface between trabecular and cancellous bone can be mimicked to some extent as different density and porosity values can be easily obtained by the printing of a proper design. 

**Figure 6:**
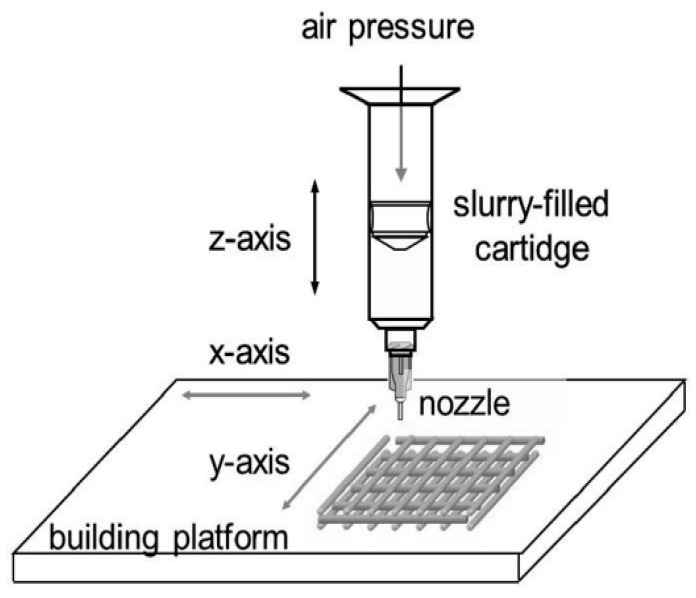
Schematic representation of a robocasting system. Picture reproduced from Gmeiner et al. [57], under a Creative Commons Attribution license (https://creativecommons.org/).

The 3D printing process builds a 3D object from a CAD model, usually by adding material through a layer by layer strategy. The most popular and affordable method to produce 3D-printed BG scaffolds involves the continuous deposition of a glass-based filament onto a building platform [61]. This approach is also called robocasting or robotic deposition; a schematic representation of a 3D printer for robocasting is shown in Figure 6 [57].

The material is extruded through a nozzle by mean of pressurized air under controlled velocity and pressure. Typically, a 3D printing machine is composed of a printing head, which contains the ink-filled syringe and a building platform, on which the material is deposited. By controlling the movements of printing head and building platform, it is possible to obtain highly-regular 3D structures with a good level of accuracy, resolution and repeatability. Movement instructions are provided by a dedicated software which connects the machine to a computer. The software is able to elaborate and convert either script files (text files) or CAD files in .stl files containing the structural and morphological features of the scaffold. Some commercial 3D-printing systems were shown to be suitable to fabricate MBG-based scaffolds, such as Manugel^®^ (ISP Alginates Ltd. Waterfield, Tadworth, UK) and 4th generation 3D-Bioplotter system (EnvisionTEC, Gladbeck, Germany).

Despite 3D printing being considered the most effective additive manufacturing technology for the processing of BGs, there are some aspects to be considered in order to obtain good printing outcomes. In fact, the quality of the final scaffold is strongly dependent on the extrusion process and the characteristics of the ink used for printing. In order to ensure a good outcome of the process and reliably reproduce the given pattern, a proper setting of printing parameters is needed in accordance with the rheological properties of the ink [61]. From a qualitative point of view, it is of paramount importance to maintain the filament straight upon extrusion to avoid the distortion of the structure and to preserve the original design. Moreover, as the scaffold is produced by continuous deposition of adjacent layers, the “strength” of the ink has to be high enough to avoid the collapse of the structure, which may also result in a deformation of the tailored 3D architecture. Another common issue concerns the formation of clogs in the nozzle during extrusion, which may result in material gaps in the scaffold or even the blocking of the printing process. All these aspects should be managed by operating a strict control on the ink properties. 

The inks used for this kind of applications are usually suspensions obtained by mixing BG particles in an aqueous solution containing a polymeric binder, which is added to provide stability to the mixture [62]. Specifically, a good ink for 3D printing should exhibit the following properties [62]: (i) pseudo-plasticity, which allows the ink to flow through a small-diameter nozzle without applying high air pressure; (ii) non-flowable mass, in order to preserve the shape after deposition; and (iii) high strength, thus being able to withstand the weight of the overlying structure. Pluronic F-127 is one of the most commonly-used binders for 3D printing in bone scaffold applications [63,64,65], together with ethyl cellulose, polyethylene glycol and carboxymethyl cellulose [66,67,68,69].

One of the key aspects of 3D printing concerns the capability of the ink to change its rheological properties under the action of particular chemical or physical conditions, such as external temperature or the drying process, in order to have a consolidated and “fixed” structure [63,70]. Delivery of the ink can take place according to two different configurations. In the first case, the ink is delivered by a constant displacement of the plunger inside the syringe, where the pressure is properly modulated for maintaining such displacement constant over time. The second configuration involves the maintenance of a constant pressure inside the syringe [71]. Controlling the displacement of the plunger is considered the most effective and reliable strategy to ensure a homogeneous delivery of the material on the building platform; on the contrary, maintaining a constant pressure may be difficult due to the changes that can occur in the rheological behavior of the ink, as explained above.

The quantity of material (ink) which is deposited on the building platform can be controlled by two factors, that is, the velocity of displacement of the printing head in the xy (horizontal) plane and the dispensing pressure. The higher the velocity of displacement, the lower the amount of ink deposited at a fixed pressure value. On the contrary, the higher the pressure used to extrude the filament, the thicker the deposited filament (rod). Therefore, although these two parameters can be set separately and are independent of each other, they need to be jointly adjusted as their variations have an effect on the same result. 

Another factor to be considered is the spacing along the vertical axis (z-spacing), which runs perpendicularly to the building platform. The z-spacing determines and controls the adhesion between adjacent layers and, thus, needs to be adjusted according to some specifications like the mean thickness of the rods and the diameter of the nozzle. If the z-spacing is too low, the movement of the nozzle can destroy the printed layers by touching them, while if it is too large, the gap is too high to allow the adhesion between the layers. 

In order to allow good reproducibility of the layers and periodicity of the structural features of the scaffold, the building platform should be flat and perpendicular to the extrusion axis. It is common to use flexible substrates, such as acetate sheets, in order to facilitate the detachment of the scaffold from the substrate after drying [72,73]. 

Drying conditions should also be carefully controlled in order to avoid crack formation due to sudden water elimination and shrinkage of the structure. 3D-printed scaffolds are usually affected by inhomogeneous drying conditions from the top of the structure to the bottom, which is in contact with the printing substrate. The top of the scaffold usually dries faster than the bottom because air recirculation is partially inhibited by the contact with the substrate and by the overlying layers. As a result, the shrinkage of the structure after uncontrolled drying conditions is usually higher at the top and may result in the formation of cracks in contact with the substrate. This problem can be overcome by operating a strict control on temperature just after printing. According to the dimensions of the printed scaffold, drying may occur at either room temperature or in mild-heating conditions. For large scaffolds (above 10 mm × 10 mm × 10 mm), treatment in pre-heated ovens may be helpful to avoid drying gradients in the 3D structure.

**Figure 7:**
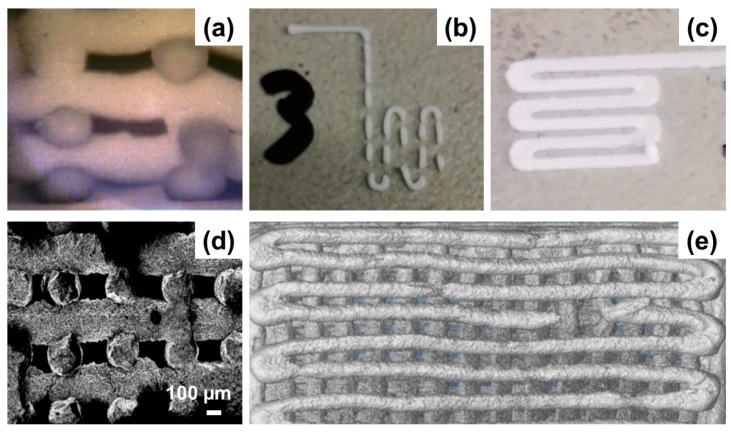
Common issues in BG scaffolds fabricated by 3D printing: collapse of the 3D structure due to the low strength of the ink (**a**); discontinuous ink deposition observed in case of low dispensing pressure and/or low printing speed (**b**); filament deformation due to low-spacing between the printing tip and the building platform and/or high dispensing pressure (**c**); defects observed in the rods of sintered scaffolds due to air-bubble entrapment in the ink during ink preparation (**d**); rod fractures after sintering (**e**). Unpublished pictures provided by the Authors.

Figure 7 summarizes the most recurrent issues related to the processing of BGs by 3D printing technology.

## 4. Application of 3D Printing to MBGs

3D printing has been successfully employed in the production of scaffolds for bone tissue engineering exhibiting hierarchical porosity. As discussed in the Section 3, under proper processing parameters, 3D printing allows a better control on morphological and structural features compared to traditional manufacturing techniques. This leads to better mechanical performances that make 3D-printed scaffolds potentially suitable even for load-bearing applications. 

Hierarchical MBG scaffolds were produced by several research groups. The great advantage of 3D printing is that the 3D structure is built following a layer-by-layer approach at room temperature, thus potentially allowing mass production of several devices characterized by structural, mechanical and morphological features varying in a very narrow range (high reproducibility). A summary of representative process parameters and synthesis details used in 3D printing of MBG scaffolds is provided in Table 1 [74,75,76,77,78,79,80,81,82].

**Figure 8:**
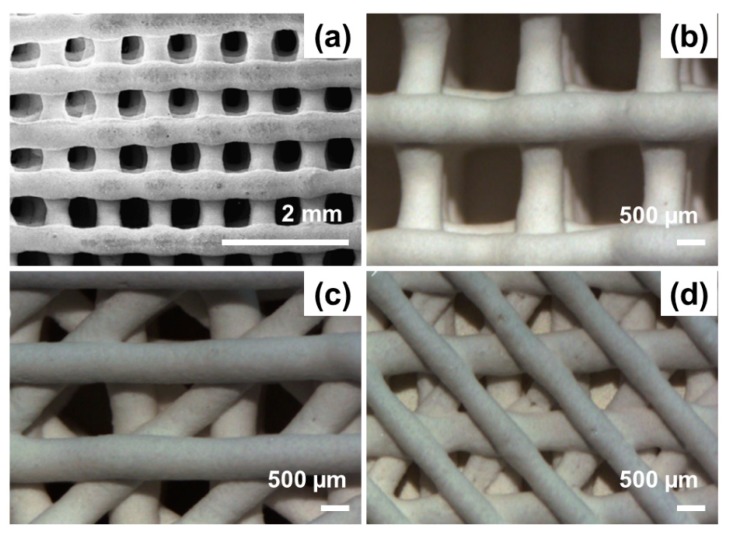
SEM images showing different macropore morphologies that can be obtained by 3D printing of MBGs: (**a**) squared, (**b**) rectangular and (**c,d**) irregular (triangular pattern) macropores. The high regularity of the pattern is well appreciable and macropore size is characterized by a narrow distribution. Pictures reproduced from Zhang et al. [77] and Wu et al. [75] with permission.

The production of several macroporous structures characterized by different macropore size and shape was extensively reported to be obtainable by operating proper modification to the text script/CAD file. Figure 8 shows that rectangular, triangular and squared macropores can be successfully obtained in 3D-printed MBG scaffolds. 

Referring to the values reported in Table 1, MBG scaffolds having macropores with size >200 µm and porosity >50 vol.% are potentially suitable for bone tissue engineering applications. In fact, these values are adequate to support proper tissue ingrowth and regeneration by allowing cell migration through the whole volume of the scaffold. Moreover, highly interconnected macropores have been demonstrated to promote the formation of a well-organized vascular network, the recirculation of body fluids and thus the diffusion of nutrients and the elimination of catabolites [83,84].

A key characteristic of MBG scaffolds is their exceptional ability to form an HA layer on their surface and struts due to the ultrahigh specific surface area (SSA), which is mainly determined by their mesoporous texture. Bioactivity and mesoporosity are intimately related since the higher the surface exposed to biological fluids, the higher the reaction rate of the material and, hence, its bioactivity [17,18,85]. N_2_ adsorption-desorption measurements are commonly used to assess SSA and mesopore size distribution by the implementation of the Brunauer-Emmett-Teller (BET) theory. MBG-based scaffolds are usually characterized by higher SSA compared to analogous systems based on melt-derived glass powders. Type-IV N_2_ adsorption-desorption isotherms (according to the classification provided by the International Union of Pure and Applied Chemistry (IUPAC) [86]), typical of mesoporous materials with narrow and ordered mesopore distribution, are usually observed [74]. SSA values of MBGs can vary within 150–400 m^2^/g depending on the mesopore-directing agent used (Table 1).

At present, there is no standardized protocol to follow for the preparation of a “good” ink with optimal rheological properties for a continuous and homogeneous extrusion of the filament. This is due to the strong interdependence that exists among ink rheological behavior, binder solution, glass composition and particle size. In general, all these parameters may be subjected to a great variation and, over time, different approaches have been experimented, each one presenting both advantages and disadvantages. The ink is usually a suspension of MBG particles into a binder/water solution. The concentration of the binder solution and the final composition of the slurry determine the properties of the ink. In this regard, sol-gel synthesis offers the possibility to obtain a slurry with suitable extrusion properties by simply acting on aging time of the sol in order to match appropriate rheological properties for printing [74].

In some preliminary studies concerning the 3D-printing of MBGs, hydroxyl propyl methylcellulose was proposed as a binding agent to confer proper rheological properties for the extrusion [74,87,88]. Garcia et al. [74] first reported the synthesis of 3D hierarchical scaffolds based on a binary SiO_2_-P_2_O_5_ MBG. A traditional sol-gel synthesis was applied for the material preparation using tetraethyl orthosilicate (TEOS) and triethyl phosphate (TEP) as SiO_2_ and P_2_O_5_ precursors, respectively. The synthesis was carried out by mixing the precursors in an ethanol/water solution in the presence of HCl (1 M) as a catalyst for the hydrolysis reaction. In order to achieve good rheological response for printing, several aging times have been tested before adding the binder (cellulosic compound) [74]. Material feed and pressure have been continuously modified during printing in order to manage viscosity variation of the ink. Hardening was carried out by keeping the scaffolds at room temperature for 24 h; then, the consolidated “greens” were sintered at high temperature to remove the organic binder (hydroxyl propyl methylcellulose) [74]. Aging period from 8 to 22 h produced a viscosity in the range of 2–3 Pa, with good flowing properties upon extrusion and rapid transformation of the ink into a solid state after contact with the building platform [74].

A dependence of MBG textural properties on the phosphorus content in the binary SiO_2_-P_2_O_5_ systems was observed—specifically, SSA was found to decrease as a result of the increase in P_2_O_5_ content compared to pure SiO_2_ samples, consistently to previous studies about the incorporation of heteroelements into the mesoporous silica network [89]. Hierarchical porosity was obtained with ordered mesopores of 4 nm, macropores in the rage 30–80 µm and ultra-large macropores of 400 µm. Degradation of organics during high-temperature sintering may lead to the formation of micro-scale porosity, which decrease the mechanical resistance of the device [74]. In order to overcome this problem, some research groups replaced methylcellulose with polyvinyl alcohol (PVA), which is known to be highly biocompatible, highly degradable and soluble in water [75]. Wu and coworkers used MBG powders (ternary glass with Si/Ca/P molar ratio of 80/15/5) to produce a suspension based on an aqueous PVA solution—in this case, scaffolds were not subjected to a sintering treatment but, after printing, were dried at low temperature and then heated up to 150 °C to thermally induce the cross-linking of PVA [75]. This approach allowed obtaining sintered scaffolds with higher mechanical properties due to the increase in PVA crystallinity as a result of the low-temperature thermal treatment. The compressive strength and elastic modulus of the scaffolds, characterized by square-pore morphology and porosity of 60.4 vol.%, were 16.10 ± 1.53 and 155.13 ± 14.89 MPa, respectively [75]. Moreover, a linear relationship between compressive strength and scaffold deformation was found for these 3D-printed scaffolds, compared to the irregular increase observed for foam-templated MBG scaffolds characterized by a jagged stress-strain curve with a maximum compressive peak of 0.08 MPa [86].

An analogous approach was also used for producing Sr-doped MBG-based scaffolds as dexamethasone (DEX)-releasing hierarchical systems. In this case, the compressive strength was about 8–9 MPa; just a small decrease of strength to 7 MPa was observed after immersion in simulated body fluid (SBF), which was attributed to the persistence of PVA with high degree of polymerization (1700 ± 50 Da), resulting in a slower dissolution of the system in aqueous solution [77]. 

Other polymers were also tested for producing MBG-containing composite scaffolds. 3D-printed hierarchical MBG/alginate composite scaffolds showed good biocompatibility, sustained drug delivery properties and HA-forming ability *in vitro*; however, the compressive strength significantly decreased from 1–1.5 MPa to 0.5–0.8 MPa after immersion for 28 days in SBF [80].

**Figure 9:**
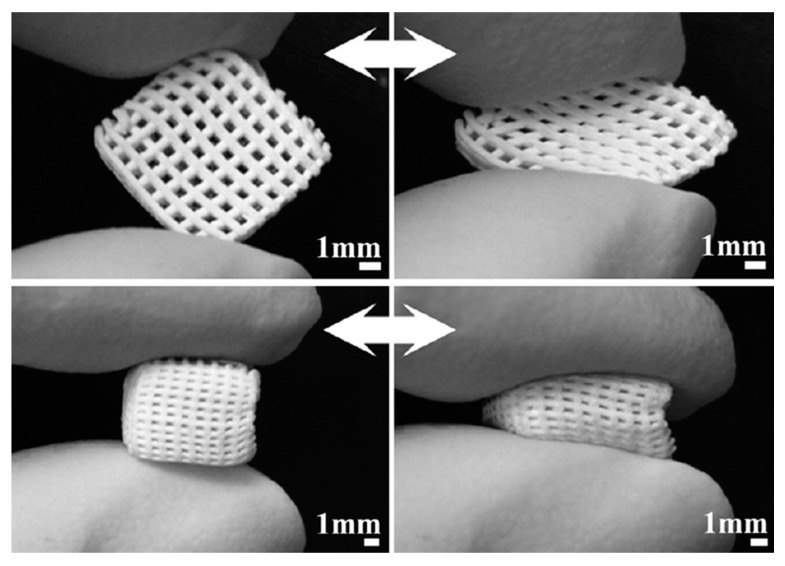
Compressibility of MBG/ polycaprolactone (PCL) composite scaffolds produced by robocasting. Pictures reproduced from Yun et al. [81] with permission.

Yun et al. [81] combined salt leaching and 3D printing to fabricate MBG/polycaprolactone (PCL) composite scaffolds exhibiting three-level multiscale porosity, including mesopores of 5 nm, mid-size macropores within 2–9 μm and large macropores around 200 μm. The scaffolds were fabricated by directly extruding the composite ink onto a chilled plate through a syringe with nozzle of 500 μm in a displacement-controlled mode. These composite scaffolds exhibited adequate compressive strength (2–4 MPa) for bone repair, good *in vitro* bioactivity and, very interestingly, a sponge-like pliable nature that may suggest a possible use in bone/cartilage interfacial tissue engineering (Figure 9) [81].

In general, as also summarized in Table 1, it is worth pointing out that the mechanical strength of 3D-printed MBG scaffolds is comparable to that of human cancellous (2–12 MPa in compression [90]), which is not achievable by using conventional fabrication methods.

## 5. Therapeutic Applications

Small amounts of doping elements can play a key role in modulating the scaffold functional properties. For example, strontium was added as a dopant to SiO_2_-CaO-P_2_O_5_ MBG compositions due to its role in combating osteoporosis (antiresorptive effect on bone, stimulation of osteoblast differentiation [91,92,93]). Interestingly, unlike what was observed for melt-derived Sr-doped BGs, the HA-forming ability was not decreased by the strontium content in Sr-containing MBG scaffolds [94], indicating that these materials were still potentially able to induce the formation of a stable bone-bonding interface. 3D-printed Sr-doped MBG scaffolds were also implanted in rat critical-sized defects [78]. *In vivo* evidence revealed that these systems, characterized by an ordered mesoporous texture, regular macroporosity (400 µm) and high porosity (70 vol.%), were able to induce bone cell adhesion, proliferation and differentiation. Moreover, such scaffolds exhibited an excellent HA-forming ability, thus ensuring primary stability to the implant and high compressive strength (about 8 MPa) for providing adequate mechanical support to the surrounding tissue.

Other therapeutic ions have also been incorporated within 3D-printed MBG scaffolds. In general, this approach is considered to be cost-effective and technologically easier compared to the use of organic biomolecules, also considering that metallic ions undergo no degradation upon manufacturing processes as may occur for thermally-degradable drugs. Shruti et al. [76] prepared 3D-printed Ce-, Ga- and Zn-doped hierarchical MBG-based scaffolds by using P123 as a mesopore SDA and cerium, gallium and zinc nitrates as precursors of the doping oxides. These dopants are known to elicit an antibacterial effect, although this specific property was not investigated in that study. The ink for 3D printing was prepared by sonication of MBG powders in dichloromethane. After that, PCL granules were dissolved in the same volume of dichloromethane under magnetic stirring. Then, the two batches were mixed together, and the so-obtained mixture was left to evaporate under continuous stirring until an injectable ink was obtained [76]. These scaffolds were found to be potentially able to induce vascularization as well as to support oxygen exchange and cell in-growth; it was also observed that textural properties and bioactive potential were successfully retained after printing. Interestingly, the HA-forming ability and textural properties of the scaffolds were comparable to those assessed for starting MBG powders. SEM morphological assessments after immersion for 7 days in SBF revealed the presence of a nanostructured HA layer characterized by the typical bone-like globular cauliflower morphology [76].

In the context of antibacterial strategies, Zhang et al. [95] used the spin-coating technique to deposit a thin layer (thickness 100 nm) of Cu-doped MBG on 3D printed β-TCP scaffolds to impart enhanced bone-forming ability and antiseptic properties due to the local release of copper ions.

Studies concerning the drug delivery ability of 3D-printed MBG scaffolds revealed a burst release of a model drug (DEX) within the first 48 h, which is particularly indicated for providing primary therapeutic action in acute inflammatory phase as soon after the device implantation [77]. A slower release was then observed, which is suitable for a long-term maintenance of the therapeutic effect. *In vivo* studies (rat model) confirmed what observed *in vitro* and reported the total depletion of the drug after 2-week implantation [78]. Simultaneous incorporation of two drugs (isoniazid and rifampin) in 3D-printed MBG scaffolds for the treatment of osteoarticular tuberculosis [82].

Zhang et al. [96] proposed the use of 3D-printed MBG/PCL composite scaffolds embedding magnetite (Fe_3_O_4_) nanoparticles in the context of cancer treatment. Specifically, the scaffolds had uniform macropores of 400 μm, porosity of 60 vol.% and compressive strength within 13–16 MPa. The incorporation of magnetite nanoparticles did not negatively affect the HA-forming property of scaffolds but endowed them with magnetic heating ability and significantly stimulated the proliferation, alkaline phosphatase activity, osteogenesis-related gene expression (RUNX2, OCN, BSP, BMP-2 and Col-1) and extra-cellular matrix mineralization of human bone marrow-derived mesenchymal stem cells. Moreover, Fe_3_O_4_/MBG/PCL composite scaffolds exhibited a sustained drug release capability for doxorubicin (DOX), used as a model anticancer drug. Therefore, these 3D-printed scaffolds showed great promise as multifunctional implants for simultaneous cancer treatment (magnetic hyperthermia combined with local drug release) and bone regeneration (enhanced osteogenic activity) at the site of tumor removal.

**Figure 10:**
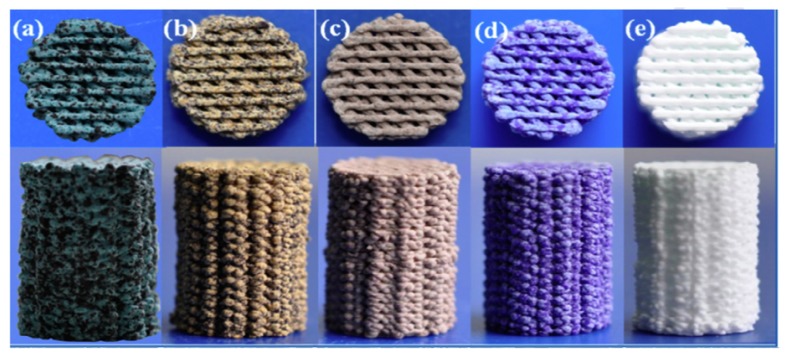
Photograph of 3D-printed scaffolds doped with different therapeutic elements: 5% Cu (**a**), 5% Fe (**b**), 5% Mn (**c**), 5% Co(**d**) and parent glass (**e**). Different colors were imparted to scaffolds by different dopants. Pictures reproduced from Liu et al. [97] with permission.

Liu et al. [97] recently reported the laser-induced photothermal properties of Cu-, Fe-, Mn- and Co-doped 3D-printed bioactive scaffolds based on sol-gel glass-ceramics for application in cancer treatment (Figure 10). After being implanted in rabbits, the materials were capable of killing tumor cells due to thermal effect, and displayed excellent bone-forming activity by stimulating the osteogenic differentiation of bone-forming cells. The combination of photothermal cancer therapy and bone regeneration represents a new strategy for the treatment of osseous tumors by using bifunctional scaffolds.

## 6. Conclusions 

The use of MBGs for the production of scaffolds by 3D printing is recognized to have a great potential in the manufacturing of bioactive synthetic bone substitutes. The clear advantages offered by additive manufacturing represent a valuable strategy to overcome the most common limitations of traditional fabrication processes for glass and ceramic scaffolds, for example, poor reproducibility and control on the final 3D structure as well as low mechanical strength. Moreover, the highly tailorable textural properties exhibited by mesoporous materials under proper synthesis conditions were found to be key in the fabrication of multifunctional systems able to simultaneously provide bone healing support and therapeutic action through the local delivery of drugs and/or biologically-active ions. Further efforts should be made in order to define standardized protocols for both the printing process and the synthesis of mesoporous materials in order to promote the extensive use of 3D-printed MBG-based scaffolds in the clinical practice, thus overcoming the current drawbacks related to the usage of biological bone grafts for the treatment of critical-sized bone defects also in load-bearing sites.

## Figures and Tables

**Table 1:** Selection of studies along with the process parameters, synthesis details and structural/textural features of 3D-printed MBG scaffolds.

Composition	Mesopore SDA	Binding Agent	Dispensing Pressure (kPa)	Printing xy Speed mm/s	Macropore Size (µm)	Porosity (vol.%)	Compr. Strength (MPa)	BET Surface Area (m^2^/g)	Ref.^a^
SiO_2_-P_2_O_5_ MBG	F-127	MC	Adapted during printing	Adapted during printing	400	40	-	152–310	[74]
SiO_2_-CaO-P_2_O_5_ MBG	P123	PVA	520–590	3	200–1300	60	~16.10	-	[75]
SiO_2_-CaO-P_2_O_5_ MBG doped with Ce, Ga and Zn	P123	PCL	-	5.3	UL > 400 MP = 100–400	-	-	398	[76]
SiO_2_-CaO-P_2_O_5_ MBG doped with Sr	P123	PVA	150–380	9–12	~400	70	-	~200	[77,78]
CSH/ SiO_2_-CaO-P_2_O_5_ MBG	P123	PCL	220–360	4.5–8.2	350	66.7–68.3	4.5–12.8	356	[79]
SiO_2_-CaO-P_2_O_5_ MBG/alginate composite	P123	Alginate	180–250	15	344–415	49.5–50.8	0.5–1.5	-	[80]
SiO_2_-CaO-P_2_O_5_ MBG/PCL composite	F-127	PCL	-	10	190	75–84	-	520	[81]
Carboxylic-modified SiO_2_-CaO-P_2_O_5_ MBG	P123	PHBHHx	-	-	~250	-	3.15	63–330	[82]

^a^ Reference numbering in the table follows the numbering in the main text. Legend: UL: Ultra Large Pores; MP: Macropores; CSH: Calcium Sulfate Hydrate; MC: methyl cellulose; PCL: poly(caprolactone); PVA: poly(vinyl alcohol); PHBHHx: poly(3-hydroxybutyrate-co-3-hydroxyhexanoate).
